# ASAS definition for sacroiliitis on MRI in SpA: applicable to children?

**DOI:** 10.1186/s12969-017-0159-z

**Published:** 2017-04-11

**Authors:** Nele Herregods, Joke Dehoorne, Filip Van den Bosch, Jacob Lester Jaremko, Joke Van Vlaenderen, Rik Joos, Xenofon Baraliakos, Gaëlle Varkas, Koenraad Verstraete, Dirk Elewaut, Lennart Jans

**Affiliations:** 1 Department of Radiology and Medical Imaging, Ghent University Hospital, De Pintelaan 185, 9000 Ghent, Belgium; 2 Department of Pediatric Rheumatology, Ghent University Hospital, De Pintelaan 185, 9000 Ghent, Belgium; 3 Department of Rheumatology and VIB Inflammation Research Center, Unit for Molecular Immunology and Inflammation, Ghent University Hospital, De Pintelaan 185, 9000 Ghent, Belgium; 4 Department of Radiology & Diagnostic Imaging, University of Alberta Hospital, 8440-112 Street, Edmonton, T6G 2B7 AB Canada; 5 Rheumazentrum Ruhrgebiet, Ruhr-University Bochum, Claudiusstr. 45, 44649 Herne, Germany

**Keywords:** ASAS definition, Sacroiliitis, MRI, Juvenile spondyloarthritis

## Abstract

**Background:**

The Assessment of Spondyloarthritis International Society (ASAS) definition for a ‘positive’ Magnetic Resonance Imaging (MRI) for sacroiliitis is well studied and validated in adults, but studies about the value of this definition in children are lacking. The aim of this study is to evaluate whether the adult ASAS definition of a positive MRI of the sacroiliac joints can be applied to children with a clinical suspicion of Juvenile Spondyloarthritis (JSpA).

**Methods:**

Two pediatric musculoskeletal radiologists blinded to clinical data independently retrospectively reviewed sacroiliac (SI) joint MRI in 109 children suspected of sacroiliitis. They recorded global impression (sacroiliitis yes/no) and whether the adult ASAS definition for sacroiliitis was met at each joint. This was compared to gold-standard clinical diagnosis of JSpA. Additionally, MRI were scored according to’adapted’ ASAS definitions including other features of sacroiliitis on MRI.

**Results:**

JSpA was diagnosed clinically in 47/109 (43%) patients. On MRI, sacroiliitis was diagnosed by global assessment in 30/109 patients, of whom 14 also fulfilled ASAS criteria. No patients with negative global assessment for sacroiliitis fulfilled ASAS criteria. Sensitivity (SN) for JSpA was higher for global assessment (SN = 49%) than for ASAS definition (SN = 26%), but the ASAS definition was more specific (SP = 97% vs. 89%). Modifying adult ASAS criteria to allow bone marrow edema (BME) lesions seen on only one slice, synovitis or capsulitis, increased SN to 36%, 32% and 32% respectively, only slightly lowering SP. Including structural lesions increased SN to 28%, but lowered specificity to 95%.

**Conclusion:**

The adult ASAS definition for sacroiliitis has low sensitivity in children. A pediatric-specific definition of MRI-positive sacroiliitis including BME lesions visible on one slice only, synovitis and/or capsulitis may improve diagnostic utility, and increase relevance of MRI in pediatric rheumatology practice.

## Background

Juvenile spondyloarthritides (JSpA) have some overlap with adult spondyloarthritis (SpA) but also important differences. JSpA are a group of related inflammatory diseases characterized by enthesitis and arthritis with a strong association to human leukocyte antigen (HLA) B27 [1]. The International League of Associations for Rheumatology (ILAR) classification of juvenile idiopathic arthritis (JIA) includes seven subtypes [2], in which JSpA is represented in enthesitis-related arthritis (ERA), psoriatic arthritis (PsA) and some types of undifferentiated arthritis. In contrast to adult spondyloarthritis (SpA), JSpA generally presents with peripheral arthritis and enthesitis of the lower extremities in the early years of the disease, while involvement of the sacroiliac (SI) and spinal joints typically occurs later [1–3].

In adults, early diagnosis of SpA has become more important to rheumatologists as new therapeutic options have become available to treat inflammation and potentially delay progression of the disease [4–7]. Increasingly, MRI of the sacroiliac (SI) joints is obtained for early detection of inflammatory changes [6], as it shows active inflammatory and structural lesions of sacroiliitis, long before radiographic changes become evident [8–11]. The Assessment of Spondyloarthritis International Society (ASAS) criteria are widely used for classification of adult spondyloarthritis [10], with the presence of active sacroiliitis on MRI as a key criterion for disease classification in the imaging arm of the ASAS criteria [10, 12–15]. A clear definition of active sacroiliitis on MRI has been defined and recently updated by the ASAS working group [10, 12].

Unlike in adult spondyloarthritis, classification of the pediatric population remains challenging. In children, many different classification systems have been proposed [2, 6, 10, 16], however none include imaging as a criterion. In adult classification systems, such as the ASAS and New York criteria, imaging plays a key role [3, 10, 14]. Therefore, adult classification criteria have been applied to children as well. An overall classification system that effectively stratifies JSpA into categories of similar clinical and prognostic implications remains elusive. Unlike in adults [12, 13, 17], the value of MRI assessment of sacroiliitis in children is not well studied, although recent studies have postulated the usefulness of MRI in JSpA [11, 18–23]. In daily clinical practice, children are increasingly being referred for MRI of the SI joints. Weiss et al. found active but asymptomatic sacroiliitis on MRI at diagnosis in JSpA, suggesting a potential role for MRI in JSpA [23]. A clear definition for a positive MRI for sacroiliitis in children is needed as much as in adults.

The ASAS definition for a ‘positive’ MRI for sacroiliitis is well studied and validated in adults [13, 24], but studies about the value of this definition in children are lacking. The aim of this study is to evaluate whether the adult ASAS definition of a positive MRI of the sacroiliac joints can be applied to children with a clinical suspicion of JSpA.

## Methods

### Patients

A retrospective study of all pediatric MRI of the sacroiliac joint from September 2013 to November 2015 was approved by the institutional ethics committee of the Ghent University Hospital. Informed consent was obtained from all parents and children. All patients were sent from the pediatric rheumatology department in a tertiary care center and were referred for MRI of the SI joints with sacroiliac joint tenderness or clinical (inflammatory) back pain (IBP) suspected for sacroiliitis in the expert opinion of the pediatric rheumatologists. As there is no established definition of IBP symptoms [25] associated with inflammatory spinal disease in children, we defined IBP as a history of back pain for at least three months with either (not and): insidious onset or improvement with exercise or no improvement with rest or pain at night (with improvement upon getting up). Sacroiliac tenderness was defined as tenderness upon palpation of the sacroiliac joint. Patients were only included when age at onset of the disease was < 16 years. The gold standard was the clinical diagnosis of JSpA made by expert opinion of our two pediatric rheumatologists (JD and RJ, respectively 21 and 35 years of experience), who reassessed the clinical files and were blinded to MRI results. They recorded from the clinical files if patients fulfilled the ILAR criteria, and diagnosed JSpA in consensus. Patients with ERA, PsA and IBD-related arthritis were considered positive for JSpA.

### Magnetic resonance imaging

MRI was performed on a body flexed array coil in a 1.5 Tesla MRI unit (Avanto, Siemens Medical, Erlangen, Germany). Sequence protocol included: semicoronal (along long axis of the sacral bone perpendicular to the second sacral (S2) vertebral body) T1-weighted (T1) turbo spin echo (TSE) (slice thickness (ST): 3 mm; repetition time/echo time (TR/TE): 368/20 ms; Field of view (FOV): 320; matrix: 512 × 384; Averages: 2; Turbo Factor (TF): 3); semicoronal short tau inversion recovery sequence (STIR) (ST: 3 mm; TR/TE/Inversion Time (TI): 5030/67/150 ms; FOV: 320; matrix: 320 × 320; Averages 2; TF: 7); axial STIR (ST: 5 mm; TR/TE/TI: 7540/67/150 ms; FOV: 400; matrix: 320 × 320; Averages: 1; TF: 7). Contrast-enhanced pulse sequences were also obtained: semicoronal (ST: 3 mm; TR/TE: 558/20 ms; FOV: 320; matrix: 512 × 384; Averages: 2; TF: 3) and axial fat-saturated T1-weighted TSE (ST: 5 mm; TR/TE: 558/9,8 ms; FOV: 350; matrix: 512 × 288; Averages: 2; TF: 3) 120 s after intravenous administration of Gadolinium-DTPA(Gd) contrast (T1/Gd) (Dotarem, 0.1 mmol/kg body weight).

### Image review

MR images were reassessed and reviewed separately by two pediatric musculoskeletal radiologists (NH, LJ), with 12 and 13 years of experience, blinded to all clinical information except age and sex.

First, a global diagnostic assessment was made as to whether the MRI was positive for sacroiliitis or not (sacroiliitis yes/no). To make this global assessment, radiologists first considered multiple active and structural features of sacroiliitis on MRI [8, 10, 11, 21].

The MRI features of active disease included bone marrow edema (BME), retro-articular enthesitis, capsulitis and synovitis. Features were scored as positive as follows: BME if periarticular high STIR signal was present in the subchondral bone in ilium or sacrum; retro-articular enthesitis if there was high STIR signal and/or enhancement of the retro-articular entheses representing soft tissue inflammation; capsulitis if high STIR signal and/or enhancement involved the SI joint capsule; synovitis was defined as presence of a hyperintense, linear signal in the joint space on T2-weighted/STIR images (which on T2 images alone is not distinguishable from fluid in the joint space, but post-gadolinium the region of synovitis will enhance, unlike fluid) and/or an enhancing tissue in the synovial part of the joint post-gadolinium administration, with signal intensity similar to vessels [8, 10, 11, 21, 26].

The MRI features implying structural damage from sacroiliitis consisted of sclerosis (lower subchondral signal than normal on all sequences), erosions (irregularities in the osteochondral interface involving both contour and signal on both T1-weighted and STIR images), fat infiltration (higher T1 signal than expected in periarticular bone) and ankylosis (continuous signal bridging all or a portion of an SI joint) [10, 11, 21]. A hazy delineation of the subchondral bone plate was considered to represent normal maturation. Each radiologist then synthesized all the MRI features of active disease and structural damage and formed a global impression as to whether sacroiliitis was present.

Next, each MRI was scored (yes/no) according to the adult ASAS definition of a positive MRI for sacroiliitis [10, 12]: “Bone marrow edema (BME) on T2-weighted sequences or bone marrow enhancement on a T1-weighted fat suppressed sequence is clearly present and located in the typical subchondral or periarticular areas. MRI appearance must be highly suggestive for SpA. If there is only one site of BME, this should be present on at least two consecutive MRI slices. If there is more than one signal present on a single slice, one slice may be enough.”[12] Other MRI features representing active inflammation of the SI joint such as enthesitis or capsulitis, or structural lesions alone are not sufficient for a ‘positive’ MRI for sacroiliitis. If an inflammatory bone marrow lesion appears to be present but it is hard to determine whether the lesion meets the criterion ‘highly suggestive for SpA’, then the decision may be influenced by the presence of concomitant structural damage and or other signs of inflammation, which in themselves do not suffice to meet the criterion” [12].

We also tested several alternative ‘adapted’ ASAS definitions for a positive MRI for sacroiliitis, which each used the same definition as above except that: 1. a study is positive even if BME was only seen on one slice or location; or 2. synovitis is present; or 3. capsulitis is present; or 4. retroarticular enthesitis is present; or 5. Any of the structural lesions from sacroiliitis are present; or 6. BME is seen in only one slice or location AND/OR synovitis is present (i.e., a combination of ‘adapted’ definitions 1 and 2); or 7. BME is seen in only one slice or location AND/OR capsulitis is present (i.e., a combination of ‘adapted’ definitions 1 and 3); or 8. BME is seen in only one slice or location AND/OR synovitis is present AND/OR capsulitis is present (i.e., a combination of ‘adapted’ definitions 1,2 and 3).

### Statistical analysis

Statistical analysis was performed using SPSS 20.0 software for Windows (SPSS, Chicago, IL, USA). Basic descriptive statistics for categorical data were recorded. The diagnostic utility for clinical diagnosis of JSpA was determined by using decision matrix analysis, generating sensitivity (SN) and specificity (SP) for the global diagnostic impression, ASAS definition and 8 adapted versions of the ASAS definition, each compared to the clinical gold standard diagnosis of JSpA. The likelihood ratio of a positive test (LR+) was calculated as sensitivity/(1-specificity) and the likelihood ratio of a negative test (LR-) value was calculated as (1-sensitivity)/specificity.

Interobserver agreement between the radiologists was calculated using kappa (κ) statistics. The levels of agreement were considered to be slight, fair, moderate, substantial, near-perfect and perfect at κ values of respectively 0–0.20, 0.21–0.40, 0.41–0.60, 0.61–0.80, 0.81–0.99 and 1.00 [27].

## Results

### Demographics

Of the 109 children, 41 (38%) were boys, 68 (62%) were girls. The mean age of our population was 13.6 years (range 6.8–17.9). HLA-B27 was positive in 31/109 (28%) patients, and negative in 49 (45%). In 29 (27%) patients, HLA-B27 was not obtained by the referring clinician, since this test was not specified to be mandatory in our MRI study design. IBP was reported in 95 patients, SI joint tenderness in 45 patients. In 31 patients, both of these symptoms were present. JSpA was clinically diagnosed in 47 (43%) patients according to the expert opinion of our pediatric rheumatologists, 45 of these children met the ILAR criteria for ERA (*N* = 36) or PsA (*N* = 9). Two patients with inflammatory bowel disease (IBD) and arthritis were also classified as JSpA. Other diagnoses were oligoarticular JIA (*N* = 8), polyarticular JIA (*N* = 2), mechanical pain (*N* = 41), hyperlaxity syndrome (*N* = 4), Behçet disease (*N* = 2), Chronic Recurrent Multifocal Osteomyelitis (CRMO) (*N* = 1), auto-inflammatory syndrome (*N* = 1), reactive arthritis (*N* = 1), psoriasis (*N* = 1) and hyperexcitability (*N* = 1). One patient was diagnosed with lymphoma and was excluded from the study.

Demographics for patients with and without JSpA are summarized in Table 1.

**Table 1:** Demographics of the study population

	Patients with JSpA		Patients without JSpA	
	(*N* = 47)		(*N* = 62)	
	*N* (%)		*N* (%)	
	MRI +	MRI –	MRI +	MRI –
	(*N* = 23)	(*N* = 24)	(*N* = 7)	(*N* = 55)
	N (%)	N (%)	N(%)	N (%)
Age (years)	10.8–18 (mean 15.0)	7.7–17.1 (mean 12.6)	12.7–18,8 (mean 15.1)	6.8–18 (mean13.1)
Male	13 (57%)	12 (50%)	1 (14%)	15 (27%)
HLA-B27 +	13 (27%) (0 ND)	8 (33%) (2 ND)	2 (29%) (1 ND)	8 (15%) (25 ND)
Inflammatory back pain	20 (87%)	18 (75%)	7 (100%)	50 (91%)
Sacroiliac joint tenderness	13(57%)	13 (54%)	3 (43%)	16 (29%)
IBP AND sacroiliac joint tenderness	10 (43%)	7 (29%)	3 (43%)	11 (20%)
Arthritis (peripheral)	13 (57%)	16 (67%)	0 (0%)	13 (24%)
Enthesitis (peripheral)	14 (61%)	15 (63%)	1 (14%)	5 (9%)

(*N* Number of patients, *JSpA* Juvenile Spondylarthropathy, *MRI+* sacroiliitis on MRI according to global assessment), *MRI -* normal MRI, *HLA-B27* Human Leukocyte Antigen B27, *ND* Not determined)

### MRI assessment

**Fig. 1:**
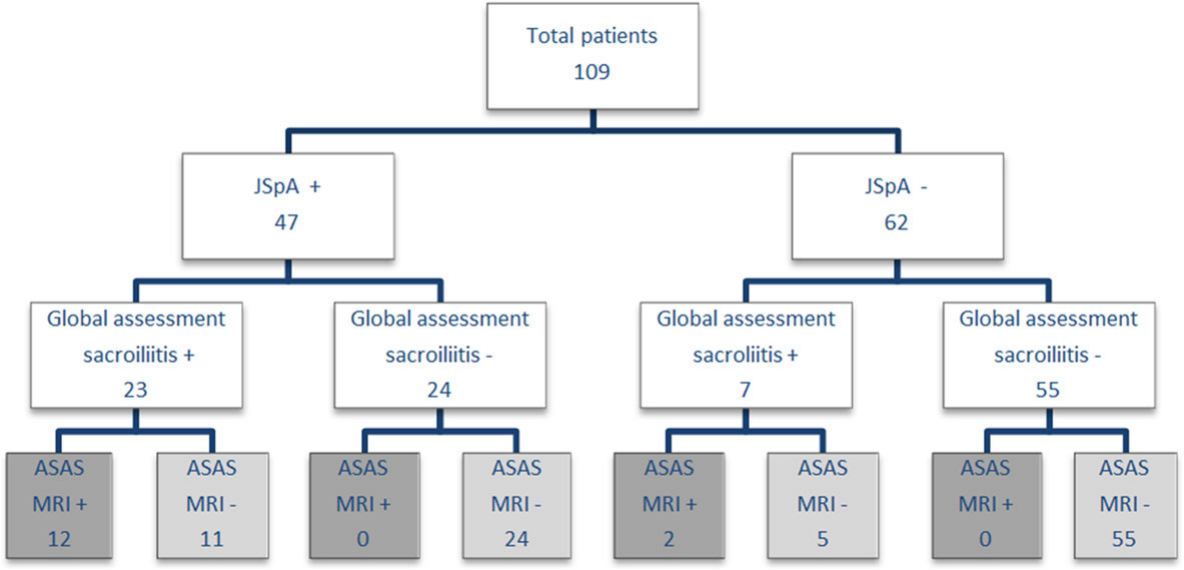
Flow chart showing the number of patients with and without sacroiliitis according to MRI global diagnostic assessment of sacroiliitis and according to the ASAS definition for a positive MRI for sacroiliitis, both correlated with the final clinical diagnosis of JSpA. (MRI = Magnetic Resonance Imaging; JSpA + = patients with Juvenile Spondyloarthritis; JSpA -: patients without Juvenile Spondyloarthritis; Global assessment sacroiliitis +: one or more features of sacroiliitis present on MRI; global assessment sacroiliitis -: no features of sacroiliitis seen on MRI; ASAS MRI + = sacroiliitis present on MRI as defined by the Assessment of Spondyloarthritis International Society; ASAS MRI -: no sacroiliitis on MRI according to the Assessment of Spondyloarthritis International Society definition)

**Fig. 2:**
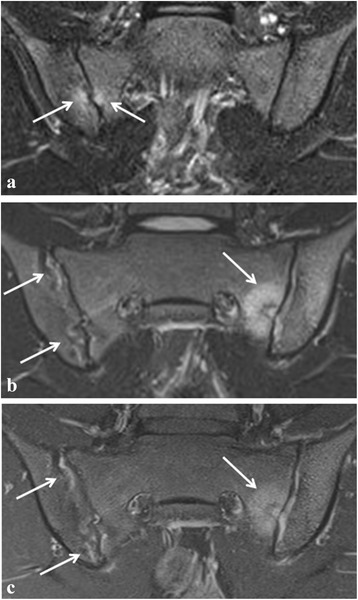
Active sacroiliitis in a 16-year-old girl with juvenile spondyloarthritis according to global assessment as well as to the ASAS definition of a positive MRI for sacroiliitis. **a** Semicoronal STIR image shows two small, focal spots of BME at the sacral and iliac side of the right sacroiliac joint (arrows). **b** Follow-up MRI 6 months later shows more extensive active sacroiliitis with bilateral high signal in the joint space and an active lesion with surrounding BME at the sacral side of the left sacroiliac joint (arrows). **c** Corresponding semicoronal fat-saturated T1-weighted image of the follow-up MRI shows bilateral enhancement of the synovium (synovitis) and of the active lesion on the left side (arrows)

**Fig. 3:**
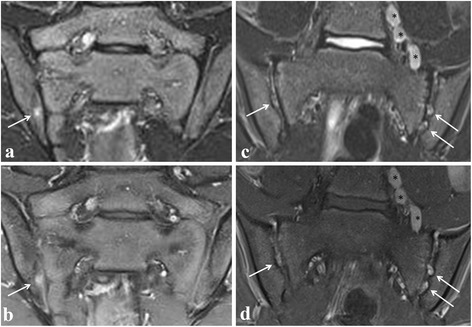
Active sacroiliitis in a 14-year-old (left) and 13-year-old (right) boy with juvenile spondyloarthritis according to a global assessment of MRI for sacroiliitis, not according to the ASAS definition of a positive MRI for sacroiliitis. Semicoronal STIR (**a** and **c**) and contrast-enhanced fat-saturated T1-weighted (**b** and **d**) images showing on the left side a focal enhancing BME lesion (seen on only one slice) at the iliac side of the right sacroiliac joint (arrows), and on the right side showing bilateral multiple enhancing spots of nodular high signal in the joint space, representing active erosions (arrows). No BME is seen. Note also the enlarged para-iliacal lymph nodes (asterisks)

**Fig. 4:**
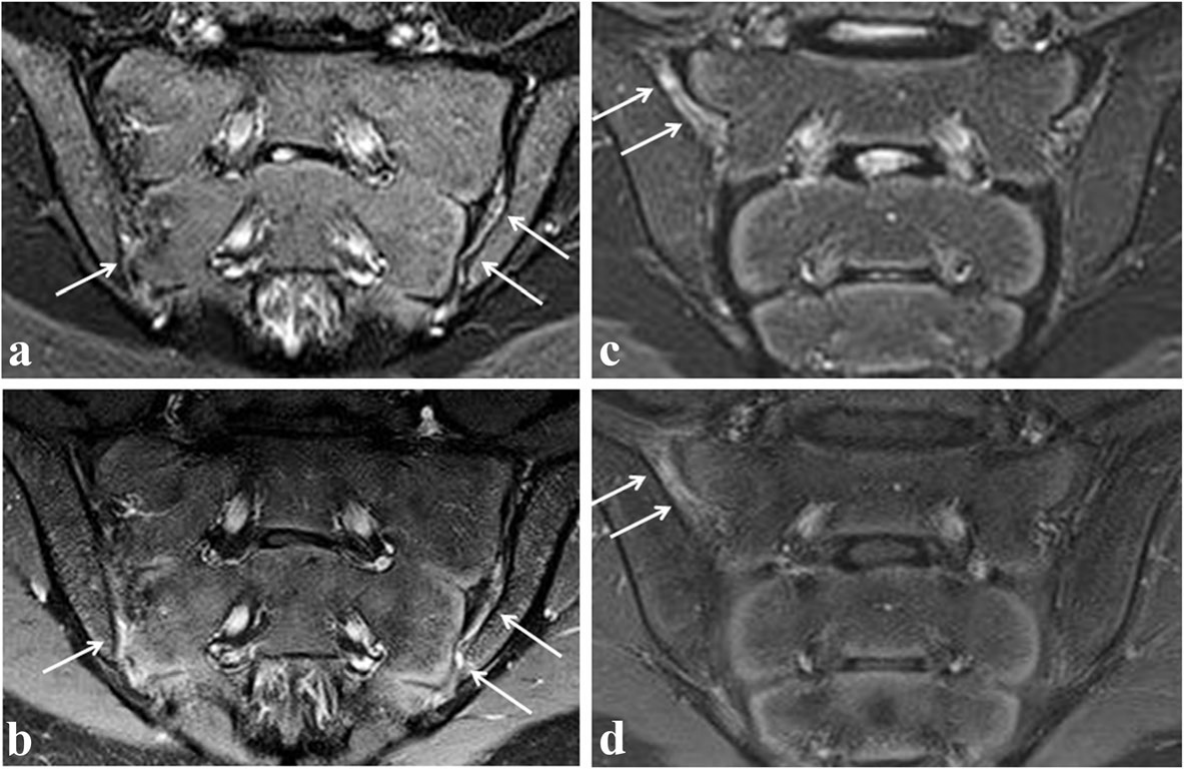
Active sacroiliitis in a 14-year-old girl (left) and a 14-year-old boy (right) with spondyloarthritis according to a global assessment of MRI for sacroiliitis, not according to the ASAS definition of a positive MRI for sacroiliitis. Semicoronal STIR (**a** and **c**) and contrast-enhanced fat-saturated T1-weighted (**b** and **d**) images on the left side showing synovitis in the caudal part of the left SI joint, also discrete in the caudal part of the right SI joint, seen as high signal in the joint space on STIR with corresponding synovial enhancement. Bone marrow edema is absent. On the right side, synovitis/retro-articular enthesitis is shown at the right sacroiliac joint (arrows). No BME is seen

MRI diagnosis of sacroiliitis according to global assessment and the adult ASAS definition for a positive MRI for sacroiliitis are summarized in Fig. 1. All patients with a positive MRI according to the ASAS definition were positive according to a global assessment for sacroiliitis (Fig. 2). In 16 of 30 patients considered to have sacroiliitis according to global assessment, the ASAS criteria for a positive MRI for sacroiliitis were not fulfilled (Figs. 3 and 4). The most frequent features which the radiologists considered to indicate sacroiliitis according to global assessment in these 16 patients are BME only seen on one slice or location, synovitis and capsulitis. In 11/16 patients, features of sacroiliitis other than BME (synovitis, capsulitis, retroarticular enthesitis of structural lesions) are seen in the absence of BME (Fig. 3 cd – Fig. 4).

### Diagnostic utility of MRI

MRI results according to global diagnostic assessment of sacroiliitis, according to the ASAS definition for a positive MRI for sacroiliitis, and according to the ‘adapted’ definitions (including small BME lesions visible on one slice only, capsulitis, retro-articular enthesitis, synovitis or structural lesions, and combinations of these features) are correlated with the final clinical diagnosis of JSpA. Sensitivity (SN), specificity (SP), positive (LR+) and negative likelihood ratio’s (LR-) for diagnosis of JSpA are shown in Table 2.

**Table 2:** The sensitivity, specificity, positive and negative likelihood ratios for diagnosis of JSpA (clinical gold standard) predicted by MRI global assessment, by the ASAS definition of a positive MRI and for ‘adapted’ ASAS definitions, including BME lesions only seen on one slice or location, synovitis, capsulitis, retro-articular enthesitis, structural lesions and combinations of these features

	N	Sensitivity	Specificity	LR	LR
		% (95% CI)	% (95% CI)	+	-
Global assessment	30	49 (34.1–63.9)	89 (78.1–95.3)	4.45	0.57
ASAS definition	14	26 (13.9–40.4)	97 (88.8–99.6)	8.67	0.76
ASAS OR focal lesions	20	36 (22.7–51.5)	95 (86.5–99.0)	7.20	0.67
ASAS OR synovitis	20	32 (19.1–47.1)	92 (82.2–97.3)	4	0.74
ASAS OR capsulitis	17	32 (19.1–47.1)	97 (88.8–99.6)	10.67	0.70
ASAS OR retroarticular enthesitis	18	30 (17.3–44.9)	94 (84.3–98.2)	5	0.74
ASAS OR structural	16	28 (15.6–42.6)	95 (86.5–99.0)	5.60	0.76
ASAS OR focal lesions OR synovitis	26	43 (28.3–57.8)	90 (80.1–96.4)	4.30	0.63
ASAS OR focal lesions OR capsulitis	23	43 (28.3–57.8)	95 (86.5–99.0)	8.60	0.60
ASAS OR focal lesions OR synovitis OR capsulitis	28	47 (32.1–61.9)	90 (80.1–96.4)	4.7	0.59

(*N* Number of sacroiliitis-positive patients, *95% CI* 95% confidence Interval, *LR+* positive likelihood ratio, *LR* - negative likelihood ratio, *ASAS* Assessment of Spondyloarthritis International Society)

When we included BME lesions that are only visible on one slice in the definition, the sensitivity (SN) rose from 26% to 36%, with minimal lowering of specificity (SP) (from 97% to 95%) (Table 2). Including both BME lesions seen on one slice and synovitis in the definition, resulted in a higher SN of 43%, unfortunately lowering SP from 97% to 90%. Including BME lesions seen on one slice and capsulitis in the definition, resulted in a higher SN of 43%, with only minimal lowering of the SP from 97% to 95%. Including BME lesions seen on one slice, synovitis and capsulitis in the definition, resulted in a higher SN of 47% - almost as high as for global assessment (49%) – at the cost of a lower SP (90%), also comparable to SN for global assessment (89%).

### Interobserver agreement

Although the radiologists worked blinded to each other’s readings, there was almost perfect inter-observer agreement (κ = 0.98) for the global assessment, and perfect agreement for the ASAS definition (κ = 1). In one case, radiologists were inconclusive for global assessment, and diagnosis of sacroiliitis on MRI in this case was made in consensus.

## Discussion

Our study is the first assessing the value of the ASAS definition of a positive MRI for sacroiliitis in children. Currently, there is no clear definition of a ‘positive’ MRI for sacroiliitis in JSpA.

Recently, Weiss et al. evaluated the prevalence of sacroiliitis and the accuracy of physical examination and back pain to detect sacroiliitis, using pelvic MRI in 40 children with newly diagnosed JSpA [23]. Active sacroiliitis on MRI was defined according to the ASAS definition [10, 12]. They concluded that active sacroiliitis on MRI is common at diagnosis in juvenile SpA and frequently asymptomatic [23]. In 2014, Lin et al. studied 50 children with known or suspected JSpA [20]. In their study, The MRI findings of sacroiliitis were defined as described by Rudwaleit et al. [28] and included the presence of synovial enhancement, bone marrow edema, and/or erosions. However, neither their definition nor the ASAS definition of sacroiliitis on MRI have been validated in children. Furthermore, in adult SpA, it has been controversial whether presence of a structural lesion such as an erosion represents a "positive" MRI when BME is not present [10, 12, 28]. This situation is uncommon in pediatric patients, who generally present with either normal SI joint or signs of active disease, while few established structural lesions are seen until the late teens, and those that are visible are generally still active [21]. Also, Weiss and Lin both only studied children with JSpA, while in our study, all children with a history of inflammatory low back pain (IBP) and/or sacroiliac joint tenderness and were sent for MRI of the SI joints were included.

In our study, we compared the radiologist global assessment of MRI for sacroiliitis with the ASAS definition of a positive MRI in a cohort of children with IBP and/ or sacroiliac joint tenderness, regardless of their final diagnosis. MRI global assessment for sacroiliitis had similar diagnostic yield for JSpA as in adult studies, with slightly lower sensitivity (49%) and similar specificity (89%) compared to Aydin et al. and Weber et al. [13, 24]. The ASAS definition for MRI-positive sacroiliitis had a much lower sensitivity for JSpA in children than in adults (26% vs. 67–79%) and was more specific in children (97% vs. 88–89%) (Table 3). Moreover, we found that the sensitivity of the ASAS definition was only half as high as the sensitivity of the global assessment in our pediatric patients (26% vs 49%).

**Table 3:** Sensitivity and specificity for diagnosis of JSpA for global assessment of MRI and for the ASAS definition of a positive MRI of children compared to adults according to studies of Ayden et al. [13] and Weber et al. [24]

			Sensitivity	Specificity	LR +	LR -
ADULTS	Aydin et al. [13]	Global	66%	94%	11	0.36
		ASAS	79%	89%	7.18	0.24
	Weber et al. [24]	Global	51%	97%	17	0.51
		ASAS	67%	88%	5.58	0.38
CHILDREN	This study	Global	49%	89%	4.45	0.57
		ASAS	26%	97%	8.67	0.76

(*Global* global assessment of MRI for sacroiliitis, *ASAS* assessment of MRI according to the ASAS definition of a positive MRI for sacroiliitis)

There are several possible explanations for the lower sensitivity in children. First of all, children with JSpA most often present with peripheral arthritis and enthesitis of the lower extremities early in the disease. Axial involvement is often not seen at presentation, but may occur years later [1, 3], and in our experience, may be less extensive at presentation compared to adults [18, 21]. We observed that BME in children, when present, appears not to be as extensive as in adults, frequently insufficient to fulfill the ASAS criteria. In some cases, erosions, capsulitis or synovitis can be seen with little or no surrounding edema, a finding previously reported by Lin et al. [20], thus not fulfilling the ASAS criteria. In 6/30 of the positive MRI for sacroiliitis according to a global assessment, a small focal T2/STIR hyperintense and enhancing lesion was the only finding on MRI. All 6 patients were clinically diagnosed with JSpA by the pediatric rheumatologists, implying that the presence of small lesions in children may therefore be more important than previously suspected. However, caution is needed, as partial volume or other normal features such as vessels, cartilage, subchondral defects or osseous clefts may have a high STIR signal and thus mimic active inflammation or erosions [29]. Other potential confounders include features of natural growth including progressive ossification of the segmental and lateral apophyses of the sacral wings and variation of the width of the joint space [30]. Because of the ongoing ossification process, joint margins can look irregular and blurred, especially on T1 weighted images, making it even more difficult to evaluate erosions [21]. Müller et al. reported a high prevalence of bony depressions, signal changes suggestive of bone marrow edema and joint fluid on MRI of the pediatric wrist [31]. However, little is published about the MRI appearance of normal sacroiliac joints in children. Another factor which may contribute to the lower sensitivity for JSpA is the relatively smaller size of the SI joints in younger children, in whom the entire joint is captured in only a few 3-mm slices – BME in a single MRI slice obtained in a small child may represent a similar proportion of the joint as in more than one slice of an adult patient. Thinner slices might be a solution, but this is a technical challenge, especially for the STIR sequence, which already has a low signal to noise ratio.

We found the ASAS definition of a positive MRI for sacroiliitis to be more specific (SP = 97%) than global assessment of sacroiliitis in JSpA (SP = 89%), which is an opposite finding compared to adults, where specificity of the ASAS definition is lower compared to global assessment [13, 24]. In adults, BME is also frequently seen in patients presenting with non-rheumatological entities that clinically mimic sacroiliitis such as degenerative disease, lumbosacral transitional anomaly, spondylolysis, fracture, infection and tumor [32, 33]. As these entities, especially degenerative disease, are less frequently seen in children, specificity of BME in children increases. Furthermore, children are frequently physically active, and sacral stress injuries have been described [34]. This suggests that children might also be prone to developing BME secondary to overuse. Grampp et al. described overuse edema in the hand on MRI [35], however, there is a lack of research on this phenomenon in the pelvis. In our pediatric study, global assessment by our two radiologists was about twice as sensitive as the ASAS definition (SN = 49% vs. 26%), implying that the ASAS definition may be too strict in children.

Since specificity of the ASAS definition of a positive MRI for sacroiliitis is very high in children, improving the definition should aim to further increase sensitivity without lowering the specificity, i.e., providing a formal approach to reach at least the level of accuracy achieved by the radiologist’s global impression in this study. Simply using the global impression as MRI diagnostic standard would be problematic since this would vary by the level of experience of the MRI reader and also does not provide insight into which features of disease contribute to diagnosis.

We studied several variations of the ASAS definition. We began with the basic adult ASAS definition for sacroiliitis on MRI, and combined it with the other features of active sacroiliitis (synovitis, capsulitis, enthesitis), as well as with structural features (erosions, sclerosis, fat deposition and ankylosis) of sacroiliitis seen in children [21]. As structural lesions such as erosions are not as frequently seen in children compared to adults [9, 21], all structural features were grouped (‘+ structural’). Structural lesions are not included in the current ASAS definition, although several recent adult studies indicate that structural lesions, particularly erosions, may contribute substantially to the diagnostic utility of the definition of a positive SI joint MRI [36, 37]. In previous studies, we observed that MRI features of sacroiliitis can be slightly different in the pediatric population compared to adults [9, 18, 21]. As we noticed that BME seen in only one location or slice was the most frequent source of discrepancies, in which sacroiliitis was diagnosed by global impression but not by ASAS definition, we also tested a definition including these small lesions seen on one slice only. Synovitis and capsulitis are also seen in children, but not included in the adult ASAS definition. Finally, we also made combinations of the three most frequent features radiologists consider to indicate sacroiliitis according to global assessment without fulfillment of the ASAS criteria. Assessing all MRI features of sacroiliitis is mandatory to find the best combination of features. However, more and large, prospective, multicentric studies are needed.

Allowing single-slice BME to diagnose sacroiliitis increased SN from 26% to 36%, adding synovitis or capsulitis as individual diagnostic factors each increased SN to 43%, and allowing single-slice BME, synovitis and/or capsulitis to define presence of sacroiliitis achieved SN = 47%, SP 90%, comparable to global impression (SN = 49%, SP = 89%). Structural lesions were less useful; allowing presence of 1 or more structural lesions to diagnose sacroiliitis only slightly increased SN to 28%, also lowering specificity to 95%. The lower specificity when including structural lesions suggests that erosions have been identified by our readers in other than JSpA patients, which partly might be due to misreading. As described, erosions are difficult to assess, especially in smaller children who have irregular delineation of the joint space because of the ongoing ossification process. There still is much debate on the exact definition of sacroiliitis on MRI, and other features than BME are not included currently. According to the most recent revision of the ASAS definition, these other features increase suspicion for sacroiliitis on MRI [12]. Still, this remains difficult, especially in children, as no one knows whether sacroiliitis seen on MRI correlates to a histopathological gold standard. Using other features than just BME is likely to increase the detection rate but will also generate more false positives, which can reduce the specificity. Optimizing this requires more, larger, multicentric studies, assessing different combinations of features as we have done in this study.

Thus, in this study the optimal variation of the ASAS definition to most sensitively detect sacroiliitis with minimum decrease in specificity was to make the ASAS definition for a positive sacroiliac joint MRI less strict by including BME lesions seen on one slice, capsulitis or synovitis. The sensitivity of the ‘adapted’ definitions was comparable to expert radiologist global impression. Still, as found by others, likely due to the ‘limbs-first’ pattern of onset of many cases of JSpA, about half of cases of JSpA may be completely imaging-negative at first diagnosis by any criteria [11].

There are some limitations to our study. First, although our study was substantial in size compared to other pediatric studies, we still had a relatively small number of patients (109) compared to adult SpA studies. The patient population represented referrals from a single tertiary center; referral patterns for sacroiliitis may vary elsewhere. However, this reflects clinical practice. As this study is a retrospective study, the patient group that was reassessed partially overlaps with the group of patients of a previous prospective study. However, MRI and clinical files of all patients were reassessed. Secondly, our inclusion criteria might have been not strict enough. The definition used for IBP might have been too sensitive. However, there is no established definition for IBP in children, and in our opinion, applying the adult ASAS definition for IBP in children would be too strict as many children do not meet these adult criteria. MRI was also the only imaging technique, without consistently available radiography or ultrasound. MRI scoring was performed using yes/no decisions and not according to Berlin or Canadian scoring systems [38–40], however, these scoring systems are not validated yet in children. Furthermore, there is little published data on normal appearances of pediatric SI joints. There is a need for studies in healthy children, to determine the range of normal variation in parameters such as joint fluid, enhancement and adjacent marrow signal in normal children. Lacking such studies, there is a risk of misidentifying normal findings as pathology in pediatric SI joints. Since this was a retrospective study, not all clinical criteria were available. In 40/109 patients, HLA-B27 was not obtained by the referring clinician. It may be possible that some patients might have been classified differently if HLA-B27 was obtained. The gold standard for JSpA diagnosis was clinical expert opinion, which although based on well recognized criteria is necessarily subjective. Finally, there was no control group of age- and sex-matched children.

Interpreting the MRI features of pediatric sacroiliac joints remains a challenge, and given the importance of an early diagnosis of JSpA, more experience in this specific topic is needed. There is no clear definition of a ‘positive’ MRI in JSpA at the moment. More studies are needed to develop and refine pediatric-specific definitions of a 'positive' MRI in juvenile SpA, and to determine the role of MRI in the classification criteria for juvenile JSpA.

## Conclusion

In this study, approximately half of pediatric patients with MRI showing some features of sacroiliitis would not have been considered ‘positive’ according to the current ASAS definition of MRI-positive sacroiliitis in adults. The key feature of the adult ASAS definition limiting sensitivity appears to be the need to show BME on two slices or locations. In children, who have smaller SI joints and less mechanical sources of BME, an adapted definition positive for sacroiliitis if just one BME lesion is seen, or if synovitis or capsulitis are present, appears to maximize sensitivity with little decrease in specificity. With further confirmation in larger / multi-center studies, this could improve clinical diagnostic utility of MRI for sacroiliitis in children.

## Abbreviations

ASAS: Assessment of spondyloarthritis international society
BME: Bone marrow edema
CI: Confidence interval
CRMO: Chronic recurrent multifocal osteomyelitis
ERA: Enthesitis-related arthritis
Gd: Gadolinium DTPA
HLA-B27: Human leukocyte antigen B27
IBD: Inflammatory bowel disease
IBP: Inflammatory back pain
ILAR: International league of associations for rheumatology
JIA: Juvenile idiopathic arthritis
JSpA: Juvenile spondyloarthritis
LR-: Negative likelihood ratio
LR+: Positive likelihood ratio
MRI: Magnetic resonance imaging
PsA: Psoriasis arthritis
SI: Sacroiliac
SN: Sensitivity
SP: Specificity
SpA: Spondyloarthritis
ST: Slice thickness
STIR: Short tau inversion recovery sequence
T1/Gd: T1-Weighted post gadolinium sequence
TSE: Turbo spin echo
